# Temporal Trends and Outcomes of Amyloidosis in Korea: A 14-Year Nationwide Cohort Study

**DOI:** 10.3390/jcm15010313

**Published:** 2025-12-31

**Authors:** Mi-Hyang Jung, Hae Ok Jung, So-Young Lee, Jong-Chan Youn, Yeo Reum Kim, Hoseob Kim, Woo-Baek Chung

**Affiliations:** 1 Division of Cardiology, Department of Internal Medicine, Seoul St. Mary’s Hospital, College of Medicine, The Catholic University of Korea, Seoul 06591, Republic of Korea; 2 Catholic Research Institute for Intractable Cardiovascular Disease, College of Medicine, The Catholic University of Korea, Seoul 06591, Republic of Korea; 3 Data Science Team, Hanmi Pharm. Co., Ltd., Seoul 05545, Republic of Korea

**Keywords:** amyloidosis, epidemiology, outcome assessment, health care, mortality

## Abstract

**Background/Objectives**: The diagnostic and therapeutic landscape of amyloidosis has evolved significantly with the introduction of non-invasive imaging and novel agents. However, contemporary real-world data reflecting these recent changes—particularly regarding the burden and prognostic impact of cardiac involvement—remain limited. We aimed to evaluate up-to-date temporal trends in the incidence, prevalence, and outcomes of amyloidosis using a nationwide cohort spanning the recent era. **Methods**: Using the Korean National Health Insurance Service database, we identified 5165 patients with newly diagnosed amyloidosis. Cardiac amyloidosis was defined by the presence of heart failure, cardiomyopathy, atrial fibrillation, or pacemaker implantation. Propensity score matching was performed to compare mortality risks between patients with and without cardiac involvement. Temporal trends in outcomes were analyzed across three periods (2009–2013, 2014–2018, and 2019–2022). **Results**: The incidence and prevalence of amyloidosis steadily increased, with a marked rise observed after 2019. Cardiac involvement was identified in 44.6% of patients and was associated with significantly higher risks of all-cause death (hazard ratio [HR] 1.396; 95% CI 1.214–1.606) and cardiovascular death (HR 1.879; 95% CI 1.254–2.816) in the matched cohort. Notably, while all-cause mortality gradually declined over the study period, cardiovascular mortality showed no significant improvement, remaining persistently high even in the most recent cohort. **Conclusions**: In this contemporary nationwide cohort, the burden of amyloidosis has grown over the past decade. Despite improvements in overall survival, the persistent risk of cardiovascular mortality highlights a critical unmet need for targeted cardiovascular management in this population.

## 1. Introduction

Amyloidosis is a rare disease characterized by the deposition of amyloid fibrils in multiple organs, leading to progressive dysfunction [1,2,3]. It encompasses a heterogenous group of disorders, most notably light-chain (AL) and transthyretin (ATTR) amyloidosis, which differ significantly in pathophysiology and prognosis [2]. Despite increasing awareness of its clinical implications, amyloidosis remains underdiagnosed, often identified only at advanced stages due to limited recognition and nonspecific symptoms [2,3]. However, the landscape of amyloidosis management is rapidly evolving. Recent advancements have been pivotal across subtypes: for AL amyloidosis, the introduction of anti-CD38 monoclonal antibodies (e.g., daratumumab) has dramatically improved hematologic response and survival [2,4]. Concurrently, for ATTR amyloidosis, the widespread adoption of noninvasive imaging modalities (e.g., bone scintigraphy) and the introduction of disease-modifying therapies following the landmark ATTR-ACT trial have facilitated earlier diagnosis and improved management strategies [5,6]. Given these paradigm shifts, there is a critical need for up-to-date epidemiological data that reflects the impact of these contemporary changes on clinical outcomes.

Cardiac involvement is the most critical determinant of prognosis in amyloidosis [2,7]. The infiltration of amyloid fibrils into myocardium leads to restrictive cardiomyopathy, manifesting as heart failure with preserved ejection fraction, atrial fibrillation, and conduction system disorders requiring pacemaker implantation. Furthermore, recent studies have highlighted the frequent coexistence of cardiac amyloidosis and degenerative aortic stenosis, particularly in the elderly, necessitating careful screening in this population [8]. Historically, patients with advanced cardiac involvement, particularly in AL amyloidosis, faced a median overall survival of less than 12 months [9]. A previous population-based study from the Danish National Registry showed that while 5-year mortality for cardiac amyloidosis improved from 91.4% (1998–2002) to 68.4% (2013–2017), it remained significantly worse than non-cardiac cases [7]. Crucially, however, prior studies have not fully captured the most recent era (post-2018), during which awareness and therapeutic options have expanded most dramatically. Furthermore, few studies have directly compared the temporal trends of cardiovascular-specific mortality between patients with and without cardiac involvement in this modern era [10].

In light of recent diagnostic and therapeutic shifts, our study aimed to provide a comprehensive, updated analysis of epidemiology and outcomes using a nationwide cohort database extended through 2022. Specifically, we sought to (1) evaluate temporal trends in incidence and prevalence, (2) directly compare outcomes between patients with and without cardiac involvement, and (3) assess whether improvements in overall survival have been accompanied by reductions in cardiovascular mortality over the past 14 years.

## 2. Materials and Methods

### 2.1. Data Source

This study utilized data from the Korean National Health Insurance Service (KNHIS), a nationwide healthcare database covering the entire Korean population under the country’s single-payer, mandatory health insurance system. The KNHIS database integrates claims-based records, including demographic information, disease diagnoses (coded using the International Classification of Diseases, 10th Revision [ICD-10]), medical procedures, prescriptions, hospitalizations, and mortality data. Detailed descriptions of the KNHIS database have been published elsewhere [11,12]. The KNHIS dataset has been widely used in epidemiological research and could be a valuable resource for studying rare diseases like amyloidosis, particularly for assessing its epidemiology and clinical outcomes at a population level [13,14]. While tertiary high-volume center cohorts have provided key insights into amyloidosis in Korea [15], the geographic dispersion of patients and the rarity of the disease underscore the need for nationwide cohort studies to evaluate incidence, prevalence, and long-term outcomes. In this regard, the KNHIS database offers a unique opportunity to investigate amyloidosis at the national level.

### 2.2. Study Population

Patients with amyloidosis were identified from the KNHIS database between 2009 and 2022 using either the ICD-10 code E85 or registration in the Rare Intractable Disease (RID) program under the code V121. In Korea, the RID program provides financial support to patients with rare and intractable conditions such as amyloidosis. To ensure appropriate use of this benefit, the registration process is strictly regulated by the government, supporting the use of the RID code (V121) as a reliable diagnostic marker for identifying amyloidosis.

Cardiac amyloidosis was defined, adapted from the methodology of a previous study [7], as a diagnosis of amyloidosis accompanied by at least one of the following four criteria: (1) a diagnosis of heart failure with evidence of heart failure-related diagnostic testing (BNP, echocardiography, or cardiac magnetic resonance imaging [CMR]) within one year of the diagnosis; (2) a diagnosis of cardiomyopathy with relevant diagnostic testing (BNP, echocardiography, or CMR) within one year prior to the diagnosis; (3) a diagnosis of atrial fibrillation; or (4) a history of pacemaker implantation. The specific ICD-10 codes used are provided in Table S1. Patients who did not meet the criteria for cardiac amyloidosis were classified as having non-cardiac amyloidosis.

### 2.3. Covariates

To compare clinical outcomes between cardiac and non-cardiac amyloidosis, we balanced and further adjusted for potential covariates, including age, sex, income level, and other comorbidities (Table S1).

### 2.4. Outcomes

The primary outcome was all-cause mortality, and the secondary outcome was cardiovascular mortality. Mortality data were obtained by linking the KNHIS database with death certificate records from Statistics Korea using unique personal identifiers. All-cause mortality included any reported death, whereas cardiovascular mortality was defined as death attributed to cardiovascular diseases (ICD-10 codes I00–I99).

### 2.5. Statistical Analyses

First, we calculated the annual prevalence and incidence of amyloidosis from 2009 to 2022. Prevalent cases were defined as individuals with a diagnosis of amyloidosis in a given calendar year. Incident cases were defined as newly diagnosed patients in a given year who had no prior diagnosis. Annual prevalence and incidence rates were reported per 1,000,000 person-years. To account for temporal changes in age distribution, age-standardized prevalence and incidence rates were calculated using the direct method, with the 2005 Korean standard population as the reference. The 2005 Korean standard population is the official reference population designated by Statistics Korea and has been widely used in national epidemiological studies to ensure consistency and comparability across research. Second, we compared baseline characteristics and clinical outcomes between patients with cardiac and non-cardiac amyloidosis. Group differences were evaluated using chi-square tests for categorical variables and *t*-tests for continuous variables, as appropriate. To control for potential confounding, 1:1 propensity score matching was performed using the greedy method with a caliper of 0.001 standard deviations of the logit of the propensity score. Missing values for income level were treated as a separate category to maintain sample size and ensure covariate balance across all baselines. After matching, the balance between groups was assessed, considering a standardized mean difference in less than 0.2 as indicative of adequate covariate balance (Figure S1). Third, we evaluated overall and cardiovascular mortality in the matched cohort. Cox proportional hazards regression models were used to estimate hazard ratios (HRs) and 95% confidence intervals (CIs) for mortality outcomes. The proportional hazards assumption was evaluated using Schoenfeld residuals, with no violations detected. Given that the main analysis was conducted in a propensity score-matched cohort, crude HRs were presented as the primary results. To confirm the robustness of the findings, we also performed additional analyses adjusting for age and sex, and further adjusting for income level and comorbidities. Kaplan–Meier survival curves were generated to compare survival outcomes between groups, and differences were tested using the log-rank test. Changes in mortality over time were assessed by dividing the study period into three intervals (2009–2013, 2014–2018, and 2019–2022). All statistical analyses were conducted using SAS version 9.4 (SAS Institute, Cary, NC, USA). A two-sided *p* < 0.05 was considered statistically significant.

### 2.6. Ethics Statement

This study adhered to the Declaration of Helsinki and was approved by the Institutional Review Board on 24 March 2023 (KC23ZISI0182). The requirement for informed consent was waived due to the retrospective nature of the study and the use of anonymized data.

## 3. Results

### 3.1. Baseline Demographic Findings

Between 2009 and 2022, a total of 5165 cases of new-onset amyloidosis were identified in the present analysis. Among these individuals, 2371 (45.9%) were aged ≥65 years, and 2818 (54.6%) were male. Of the total cohort, 2306 (44.6%) had cardiac amyloidosis, while 2859 (55.4%) had non-cardiac amyloidosis. Individuals with cardiac amyloidosis were more likely to be older (aged ≥65 years: 61.0% vs. 33.7%, *p* < 0.001) and male (57.5% vs. 52.1%, *p* < 0.001) compared to those with non-cardiac amyloidosis. Additional baseline characteristics are presented in Table 1.

### 3.2. Incidence and Prevalence of Amyloidosis Between 2009 and 2022

**Figure 1:**
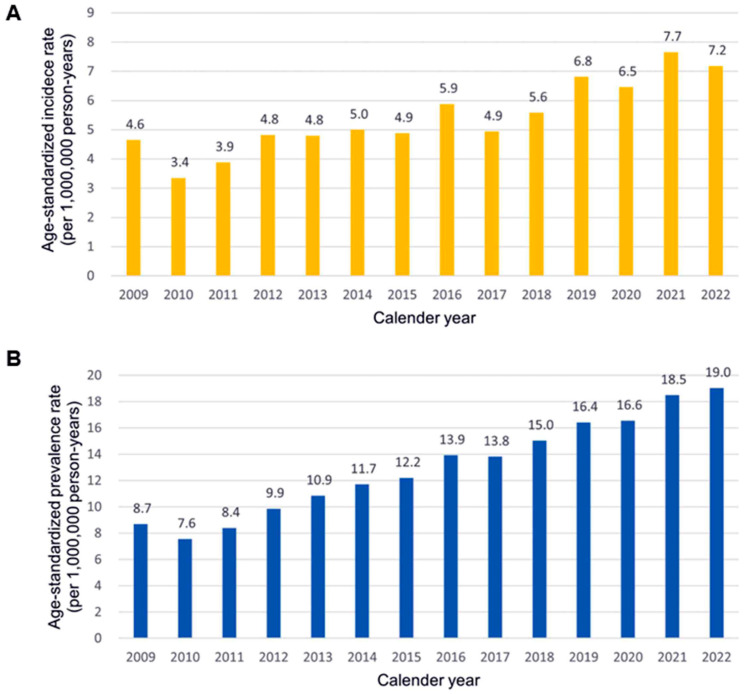
Incidence and Prevalence of Amyloidosis from 2009 to 2022 This figure presents temporal trends in the epidemiology of amyloidosis in Korea over the 14-year study period. (**A**) Age-standardized incidence rate (per 1,000,000 person-years), demonstrating a gradual increase over time, Wwith a marked rise observed after 2019. (**B**) Age-standardized prevalence rate (per 1,000,000 person-years), which steadily increased from 8.7 cases in 2009 to 19.0 cases in 2022.

Both the incidence and prevalence of amyloidosis steadily increased between 2009 and 2022 (Figure 1). In particular, the incidence rate showed a marked rise beginning in 2019. As of 2022, the age-standardized incidence rate was 7.2 per 1,000,000 person-years, and the age-standardized prevalence rate was 19.0 per 1,000,000 person-person years.

### 3.3. Clinical Outcomes in Patients with and Without Cardiac Involvement

**Figure 2:**
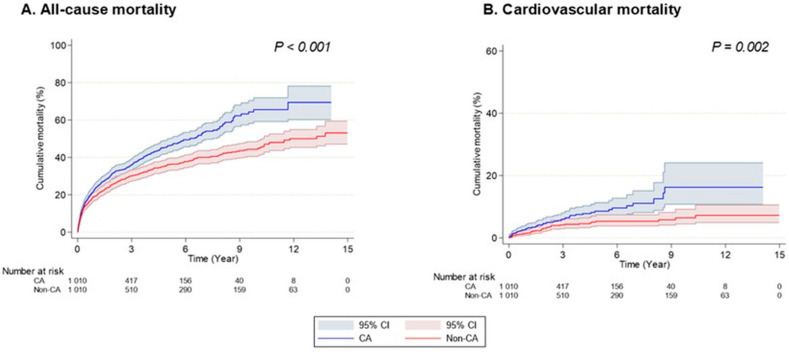
Mortality outcomes in patients with amyloidosis stratified by cardiac involvement. (**A**) Cumulative all-cause mortality. (**B**) Cumulative cardiovascular mortality. The blue line and shaded region represent patients with cardiac amyloidosis (CA), while the red line and shaded region represent those without cardiac involvement (Non-CA). Shaded areas indicate 95% confidence intervals (CIs). Patients with CA demonstrated significantly higher rates of both all-cause (*p* < 0.001) and cardiovascular mortality (*p* = 0.002) compared to the non-CA group.

After 1:1 propensity score matching, we compared 1010 individuals with cardiac amyloidosis and 1010 individuals with non-cardiac amyloidosis to assess the risk of all-cause and cardiovascular mortality. During a mean follow-up of 3.8 ± 3.4 years, a total of 810 deaths occurred. Cardiac amyloidosis was associated with a higher risk for all-cause mortality (HR 1.396, 95% CI 1.214–1.606) and cardiovascular mortality (HR 1.879, 95% CI 1.254–2.816) compared to non-cardiac amyloidosis. These associations remained after further adjustment for potential confounding factors (Table 2). Moreover, the increased risk associated with cardiac amyloidosis was consistently observed across all three time periods (2009–2013, 2014–2018, and 2019–2022). Kaplan–Meier analysis similarly demonstrated a higher cumulative incidence of both all-cause and cardiovascular mortality among individuals with cardiac amyloidosis (log-rank *p* < 0.005, Figure 2).

We further stratified the cohort based on the number of cardiac amyloidosis-defining criteria met (0, 1, 2, or ≥3). A greater number of fulfilled criteria was generally associated with an increased risk of both all-cause and cardiovascular death. The event rates for all-cause mortality were 37.0%, 40.4%, 47.9%, and 49.4% among individuals who met 0, 1, 2, and ≥3 cardiac amyloidosis-defining criteria, respectively. The association was particularly evident among individuals meeting one or two criteria, whereas those meeting three or more criteria showed a similar trend that did not reach statistical significance, likely due to the small sample size and low event count. A similar pattern was observed for cardiovascular mortality (Table S2).

### 3.4. Temporal Trends in All-Cause and Cardiovascular Mortality in Cardiac and Non-Cardiac Amyloidosis

**Figure 3:**
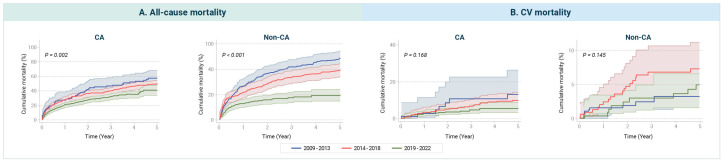
Temporal trends in mortality outcomes stratified by diagnostic period. (**A**) Cumulative all-cause mortality. (**B**) Cumulative cardiovascular (CV) mortality. The curves represent mortality trends in patients with cardiac amyloidosis (CA) and non-cardiac amyloidosis (Non-CA) across three time periods: 2009–2013 (blue lines), 2014–2018 (red lines), and 2019–2022 (green lines). Shaded areas indicate 95% confidence intervals (CIs). All-cause mortality significantly decreased over time in both the CA (*p* = 0.002) and Non-CA groups (*p* < 0.001). In contrast, no statistically significant temporal differences were observed for cardiovascular mortality in either the CA (*p* = 0.168) or Non-CA group (*p* = 0.145).

We assessed temporal trends in all-cause and cardiovascular mortality among patients with cardiac and non-cardiac amyloidosis between 2009 and 2022, with particular interest in potential differences by outcome type. During this period, all-cause mortality significantly declined in both cardiac and non-cardiac amyloidosis patients (Figure 3, Panel A). In patients with cardiac amyloidosis, cumulative all-cause mortality decreased over time, with the most recent cohort (2019–2022) showing significantly lower mortality compared to earlier periods (log-rank *p* = 0.002). A similar but more pronounced trend was observed in patients with non-cardiac amyloidosis, where all-cause mortality declined even more steeply in recent years (log-rank *p* < 0.001). In contrast, cardiovascular mortality did not show statistically significant temporal differences in either group (Figure 3, Panel B). Although the cumulative incidence of cardiovascular death appeared numerically lower in more recent cohorts, these differences were not statistically significant.

## 4. Discussion

**Figure 4:**
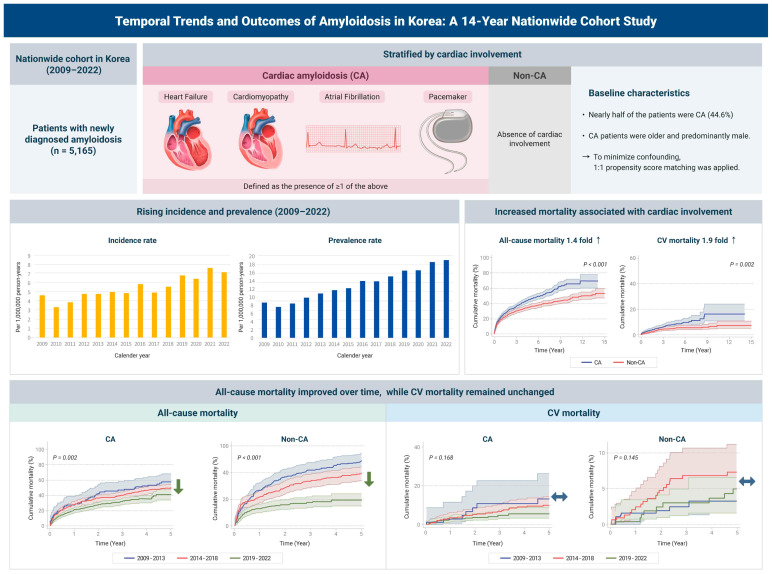
Schematic overview of the study design and major findings. This diagram summarizes the nationwide cohort construction (2009–2022) and key results. The study demonstrated a steady rise in the incidence and prevalence of amyloidosis. While cardiac involvement was consistently associated with higher mortality risks, a notable divergence in temporal trends was observed: all-cause mortality improved significantly over the study period, whereas cardiovascular mortality remained stagnant, highlighting a persistent unmet need in cardiac-specific management. CA = cardiac amyloidosis; CV = cardiovascular.

In this contemporary nationwide cohort of over 5000 patients newly diagnosed with amyloidosis between 2009 and 2022, we observed a steady increase in both incidence and prevalence, with a marked rise after 2019. Cardiac involvement was identified in approximately 45% of the cohort and was associated with a higher risk of all-cause and cardiovascular mortality compared to non-cardiac amyloidosis. The risk of adverse outcomes tended to be higher in those meeting a greater number of cardiac amyloidosis-defining criteria. Notably, while all-cause mortality gradually declined over the 14-year period, cardiovascular mortality showed no significant improvement. A schematic overview of the study design and major outcomes is presented in Figure 4.

Regarding epidemiologic trends, the age-standardized incidence rate increased approximately 1.74-fold—from 4.3 to 7.5 per 1,000,000 person-years between 2009–2013 and 2021–2022—mirroring global trends [16,17]. Although the overall incidence was slightly lower than in other countries, the temporal pattern was consistent. The temporal rise in incidence can be attributed to key factors. The steady baseline increase mirrors the rapid aging of the Korean population, as wild-type ATTR amyloidosis is an age-related condition. The sharper acceleration observed post-2019 closely aligns with increased diagnostic awareness, widespread adoption of noninvasive imaging modalities such as bone scintigraphy (introduced around 2016) [5], and heightened clinical attention following the 2018 publication of the pivotal tafamidis trial (ATTR-ACT) [6]. Although our data could not differentiate subtypes of amyloidosis, a recent study from a tertiary referral center in Italy demonstrated an exponential increase in diagnoses of ATTR amyloidosis from 2016 onward, accompanied by a steady rise in AL amyloidosis [17].

In this cohort study, cardiac involvement—defined as the presence of heart failure, cardiomyopathy, atrial fibrillation, or pacemaker implantation—was observed in nearly half of the patients with amyloidosis. In line with previous studies [7,10], cardiac involvement was associated with a poor prognosis, showing a 1.4-fold increase in all-cause mortality and a 1.9-fold increase in cardiovascular mortality. This adverse association persisted through the most recent observation period (2019–2022), underscoring the need for ongoing clinical vigilance. Furthermore, individuals meeting a greater number of cardiac amyloidosis-defining criteria demonstrated a stepwise increase in mortality risk, although the trend was not strictly linear. Among the four criteria, pacemaker implantation was paradoxically associated with decreased mortality, whereas the other three criteria—heart failure, cardiomyopathy, and atrial fibrillation—were each associated with increased risk. This suggests that while conduction system diseases is a hallmark of advanced infiltration, the intervention itself (pacing) effectively mitigates the risk of sudden bradyarrhythmic death, potentially uncoupling this specific manifestation from immediate mortality risk compared to pump failure. Nevertheless, the overall trend suggests that the greater the number of cardiac amyloidosis-defining features present, the higher the associated mortality risk.

Our study also demonstrated that overall outcomes, as reflected by all-cause mortality, have improved over time in patients with amyloidosis, regardless of cardiac involvement. As expected, cardiac amyloidosis was consistently associated with higher mortality across all time periods compared to non-cardiac amyloidosis. The decline in all-cause mortality was particularly pronounced in the most recent cohort (2019–2022), especially among those with non-cardiac amyloidosis, although a significant reduction was also observed in the cardiac amyloidosis group. The improvement in all-cause mortality, despite the lack of significant improvement in cardiovascular mortality, likely reflects a combination of factors. First, the ‘lead-time bias’ resulting from earlier diagnosis allows patients to be identified before the onset of end-stage organ failure. Second, advancements in comprehensive care have significantly reduced non-cardiovascular deaths. This includes enhanced supportive care—such as infection control, nutritional support, and volume status management—and a coordinated multidisciplinary approach to patient care. Furthermore, the growing availability of effective disease-modifying therapies for AL amyloidosis, including bortezomib-based regimens and, in selected patients, upfront autologous stem cell transplantation, has substantially improved overall survival rates [5,6,18,19,20,21]. These findings align with a previous cohort study from Denmark, which similarly reported temporal improvements in all-cause mortality among patients with cardiac amyloidosis [7].

Despite these encouraging trends in overall survival, cardiovascular mortality did not show a corresponding temporal decline. In particular, cardiovascular mortality in the 2019–2022 cohort showed only modest improvement and largely mirrored the pattern of earlier cohorts. This suggests that cardiovascular complications remain a major unresolved challenge in the management of amyloidosis, even among patients without overt cardiac involvement. Several factors may account for this persistent gap. First, cardiac amyloidosis is frequently diagnosed at an advanced stage, by which time substantial infiltration of amyloid fibrils has already occurred. Many patients also present with low blood pressure and impaired renal function, which limits the use of guideline-directed medical therapy for heart failure [3,22]. Moreover, during most of the study period, available therapies primarily focused on reducing systemic amyloid production rather than reversing or removing existing cardiac deposits. Second, although several novel therapies have been introduced in recent years, their real-world use has remained limited due to high costs and restricted access [6,23,24]. As a result, although the number of diagnosed amyloidosis cases has increased, this may not yet have translated into meaningful improvements in cardiovascular outcomes. For example, tafamidis was approved in Korea in 2020 following its FDA approval, it was not reimbursed for ATTR cardiac amyloidosis during the study period (2009–2022). Reimbursement for ATTR cardiac amyloidosis was only granted in March 2025, meaning that for the duration of this cohort study, financial barriers significantly limited patient access to this disease-modifying therapy. Similarly, while agents such as daratumumab have shown efficacy in AL amyloidosis, they were not widely available or reimbursed during most of the study period, potentially limiting their impact on the population-level cardiovascular outcomes observed in this analysis [24]. Third, as seen in the management of other malignancies, clinical care for amyloidosis often prioritizes disease-specific treatment, which may inadvertently lead to the under-recognition or under-treatment of broader cardiovascular risk factors. Routine cardiovascular health measures—such as lipid control, blood pressure management, and glucose monitoring—may be underutilized in this population, contributing to residual cardiovascular risk [25,26,27,28,29,30]. It is also worth noting that the number of cardiovascular deaths was relatively low, particularly in the most recent cohort (2019–2022) due to the shorter follow-up duration. This contributed to wider 95% CIs and limited the statistical power to detect significant temporal improvements in cardiovascular mortality, necessitating cautious interpretation of these trends. However, from a clinical perspective, the observed event rates remained stagnant, suggesting that substantial improvements in cardiovascular outcomes have yet to be realized. Consequently, the consistently flat trajectory of cardiovascular mortality, despite improvements in overall survival, underscores the need for a more targeted approach to cardiovascular care in amyloidosis.

Future research should focus on validating the efficacy of evolving therapeutic strategies. First, optimizing heart failure management remains a priority. Given that the majority of patients present with heart failure with preserved ejection fraction, the efficacy of SGLT2 inhibitors and the non-steroidal mineralocorticoid receptor antagonist finerenone—which have shown benefits in this phenotype—should be rigorously evaluated. Notably, recent evidence suggests that SGLT2 inhibitors may reduce the risk of sudden cardiac death, a finding of particular relevance to the amyloidosis population prone to fatal arrhythmias [31]. Additionally, for the subset of patients with worsening heart failure and ejection fraction < 45%, the potential role of vericiguat warrants investigation [22]. Recent consensus highlights its utility in optimizing outcomes for high-risk heart failure phenotypes, a profile that frequently overlaps with advanced cardiac amyloidosis [32]. Second, the therapeutic landscape involves distinct approaches based on amyloid subtype. For ATTR amyloidosis, the paradigm is shifting from stabilization to silencing and cure; beyond TTR stabilizers (e.g., tafamidis, acoramidis) [6,33], gene-silencing RNA therapies (e.g., vutrisiran, eplontersen) [34,35] and CRISPR/Cas9-based in vivo gene editing treatments (e.g., NTLA-2001) are emerging to halt TTR production permanently [36]. Meanwhile, for both AL and ATTR amyloidosis, fibril extraction remains a challenging but critical goal. Although recent Phase 3 trials of monoclonal antibodies targeting AL fibrils (e.g., anselamimab, birtamimab) faced setbacks in meeting primary endpoints, the concept of clearing existing myocardial deposits remains attractive [37]. Future research needs to focus on identifying responsive subgroups or developing next-generation pan-amyloid removal agents (e.g., AT-02) to overcome these hurdles [38]. As these subtype-specific and potentially distinct classes enter clinical practice, longitudinal studies spanning the next 5 to 10 years will be essential to determine whether these advancements finally translate into a reduction in the currently unchanged cardiovascular mortality rates.

Taken together, our findings suggest that while all-cause mortality is improving in the context of rising amyloidosis prevalence, cardiovascular mortality remains largely unchanged. To address this, efforts must focus on earlier diagnosis, timely therapy, expanded treatment access, and systematic cardiovascular risk management in routine care.

## 5. Limitations

Several limitations should be taken into account in the interpretation of the present findings. First, this study is based on an observational cohort design, which may limit causal inference despite rigorous statistical approaches such as propensity score matching. Specifically, quantitative clinical data such as echocardiographic measurements (e.g., ejection fraction, wall thickness) or biomarker levels (e.g., NT-proBNP) were not available in this claims database, leading to potential residual confounding. Second, the identification of amyloidosis and the classification into cardiac versus non-cardiac subtypes were based on claims data using ICD-10 codes. As administrative data are primarily collected for reimbursement rather than research, there may be discrepancies between billing codes and actual clinical conditions. Although we classified cardiac involvement using methods previously applied in a Danish cohort study [7], the possibility of misclassification remains inherent to this type of analysis. For instance, early-stage cardiac amyloidosis without overt heart failure, cardiomyopathy, or arrhythmia may have been misclassified as non-cardiac amyloidosis. Third, our cohort did not differentiate the subtypes of amyloidosis, such as AL amyloidosis, ATTR amyloidosis, or amyloid A amyloidosis. Although we attempted a sensitivity analysis to subclassify these using ICD-10 codes for hematologic malignancy and chemotherapeutic agents (e.g., bortezomib), strict reimbursement regulations in Korea during the study period limited the consistent capture of these prescriptions. This resulted in low sensitivity, rendering the approach unreliable for accurate subtype stratification in this analysis.

## 6. Conclusions

In this 14-year nationwide cohort study, the incidence and prevalence of amyloidosis in Korea steadily increased, particularly after 2019. Cardiac involvement was identified in nearly half of the patients and was consistently associated with poor prognosis. Crucially, while overall survival has improved over the past decade, the rate of cardiovascular mortality has shown little change. This divergence highlights a persistent unmet need in reducing the cardiac-specific burden of the disease. Continued efforts are essential to promote early detection, optimize treatment, and systematically manage cardiovascular risk in this growing population.

## Figures and Tables

**Table 1:** Study Population Characteristics.

Variables	Overall Population	Before Matching				After Matching			
		CA	Non-CA	*p*-Value	SMD	CA	Non-CA	*p*-Value	SMD
Total, n	5165	2306	2859	n/a	n/a	1010	1010	n/a	n/a
Age categories, years				<0.001	−0.568			0.373	0.041
<65, n (%)	2794 (54.1)	899 (39.0)	1895 (66.3)			500 (49.5)	480 (47.5)		
≥65, n (%)	2371 (45.9)	1407 (61.0)	964 (33.7)			510 (50.5)	530 (52.5)		
Sex				<0.001	0.110			0.372	−0.039
Male	2818 (54.6)	1328 (57.6)	1490 (52.1)			535 (53.0)	555 (55.0)		
Female	2347 (45.4)	978 (42.4)	1369 (47.9)			475 (47.0)	455 (45.0)		
Income level				0.001	−0.059			0.991	−0.007
Lowest tertile (0–6)	1277 (24.7)	603 (26.1)	674 (23.6)			240 (23.8)	241 (23.9)		
Middle tertile (7–13)	1249 (24.2)	524 (22.7)	725 (25.4)			223 (22.1)	224 (22.2)		
Highest tertile (14–20)	2513 (48.7)	1134 (49.2)	1379 (48.2)			525 (52.0)	521 (51.6)		
Missing	126 (2.4)	45 (2.0)	81 (2.8)			22 (2.2)	24 (2.4)		
Comorbidities, n (%)									
IHD	1372 (26.6)	929 (40.3)	443 (15.5)	<0.001	−0.575	230 (22.8)	263 (26.0)	0.087	0.076
Acute MI	212 (4.1)	151 (6.5)	61 (2.1)	<0.001	−0.218	35 (3.5)	26 (2.6)	0.242	−0.044
Heart failure	1230 (23.8)	970 (42.1)	260 (9.1)	<0.001	−0.816	176 (17.4)	165 (16.3)	0.514	−0.027
Atrial fibrillation	435 (8.4)	387 (16.8)	48 (1.7)	<0.001	−0.541	33 (3.3)	28 (2.8)	0.516	−0.018
Hypertension	3003 (58.1)	1730 (75.0)	1273 (44.5)	<0.001	−0.654	649 (64.3)	667 (66.0)	0.401	0.038
Diabetes	2163 (41.9)	1267 (54.9)	896 (31.3)	<0.001	−0.491	471 (46.6)	457 (45.2)	0.532	−0.029
Dyslipidemia	3327 (64.4)	1825 (79.1)	1502 (52.5)	<0.001	−0.585	719 (71.2)	758 (75.0)	0.057	0.085
Ischemic stroke	179 (3.5)	120 (5.2)	59 (2.1)	<0.001	−0.168	31 (3.1)	30 (3.0)	0.897	−0.005
COPD	494 (9.6)	308 (13.4)	186 (6.5)	<0.001	−0.231	63 (6.2)	76 (7.5)	0.292	0.043
CKD	1523 (29.5)	911 (39.5)	612 (21.4)	<0.001	−0.401	315 (31.2)	331 (32.8)	0.445	0.035
Multiple myeloma	637 (12.3)	498 (21.6)	139 (4.9)	<0.001	−0.510	97 (9.6)	82 (8.1)	0.240	−0.045
Carpal tunnel syndrome	257 (5.0)	161 (7.0)	96 (3.4)	<0.001	−0.164	43 (4.3)	32 (3.2)	0.196	−0.049
Lumbar spinal stenosis	1124 (21.8)	697 (30.2)	427 (14.9)	<0.001	−0.372	228 (22.6)	218 (21.6)	0.592	−0.024
All cancer	655 (12.7)	361 (15.7)	294 (10.3)	<0.001	−0.215	210 (20.8)	206 (20.4)	0.826	−0.010

CA = cardiac amyloidosis; CKD, chronic kidney disease; COPD, chronic obstructive pulmonary disease; IHD, ischemic heart disease; MI, myocardial infarction; SMD, standardized mean difference. The income status was categorized based on the tertile of all beneficiaries of the National Health Insurance Service.

**Table 2:** Clinical outcomes in the propensity score-matched population.

Outcomes	Exposure	No. of Events	Event Rate, n/1000 Person-Year	After Matching (Crude)		Age ^a^, Sex-Adjusted		Multivariable -Adjusted ^b^	
				HR (95% CI)	*p*-Value	HR (95% CI)	*p*-Value	HR (95% CI)	*p*-Value
Entire period (2009–2022)									
All-cause mortality	Non-CA	374	83.9	1 (ref.)	-	1 (ref.)	-	1 (ref.)	-
	CA	436	139.2	1.396 (1.214–1.606)	<0.001	1.351 (1.174–1.555)	<0.001	1.315 (1.142–1.515)	0.001
Cardiovascular mortality	Non-CA	40	9	1 (ref.)	-	1 (ref.)	-	1 (ref.)	-
	CA	61	19.5	1.879 (1.254–2.816)	0.002	1.767 (1.175–2.656)	0.006	1.758 (1.166–2.650)	0.007
2009–2013									
All-cause mortality	Non-CA	163	88.3	1 (ref.)		1 (ref.)		1 (ref.)	
	CA	58	146.6	1.456 (1.078–1.967)	0.014	1.356 (1.002–1.835)	0.049	1.436 (1.043–1.977)	0.027
Cardiovascular mortality	Non-CA	11	6	1 (ref.)		1 (ref.)		1 (ref.)	
	CA	11	27.8	4.203 (1.816–9.726)	0.001	3.566 (1.520–8.367)	0.004	2.915 (1.067–7.965)	0.037
2014–2018									
All-cause mortality	Non-CA	144	87.7	1 (ref.)		1 (ref.)		1 (ref.)	
	CA	205	135.7	1.438 (1.161–1.781)	0.001	1.439 (1.161–1.782)	0.001	1.426 (1.143–1.779)	0.002
Cardiovascular mortality	Non-CA	20	12.2	1 (ref.)		1 (ref.)		1 (ref.)	
	CA	29	19.2	1.491 (0.842–2.641)	0.171	1.454 (0.818–2.585)	0.202	1.391 (0.764–2.533)	0.280
2019–2022									
All-cause mortality	Non-CA	67	69.3	1 (ref.)		1 (ref.)		1 (ref.)	
	CA	173	141.2	1.962 (1.480–2.602)	<0.001	1.944 (1.466–2.579)	<0.001	1.930 (1.447–2.576)	<0.001
Cardiovascular mortality	Non-CA	9	9.3	1 (ref.)		1 (ref.)		1 (ref.)	
	CA	21	17.1	1.770 (0.811–3.866)	0.152	1.700 (0.777–3.723)	0.184	1.842 (0.827–4.103)	0.135

CA = cardiac amyloidosis; CI = confidence interval; HR = hazard ratio. ^a^ Age was treated as a continuous variable. ^b^ The multivariable model was adjusted for age, sex, income level, and comorbidities listed in Table 1. Rows are shaded to distinguish the three periods analyzed: 2009–2013, 2014–2018, and 2019–2022.

## Data Availability

The data supporting the findings of this study are available from the KNHIS. Data access is subject to review and approval by the KNHIS review committee. Re-searchers who wish to access the data can apply through the KNHIS website (https://nhiss.nhis.or.kr) after obtaining approval from their institutional review board.

## Supplementary Materials

The following supporting information can be downloaded at https://www.mdpi.com/article/10.3390/jcm15010313/s1; Figure S1: Standardized mean differences across baseline characteristics before and after propensity score matching; Table S1: International Classification of Disease (10th edition) Clinical Modification (ICD 10-CM) codes used to define study population, outcomes, and comorbidities; Table S2: Clinical outcomes based on the number of cardiac amyloidosis-defining criteria met in the propensity score-matched population.

## Abbreviations

The following abbreviations are used in this manuscript:

AL	Light-chain
ATTR	Transthyretin
CI	Confidence interval
CMR	Cardiac magnetic resonance
HR	Hazard ratio
ICD-10	International Classification of Diseases, 10th Revision
KNHIS	Korean National Health Insurance Service
RID	Rare intractable disease
