# Supplementary material for: Temporal Trends and Outcomes of Amyloidosis in Korea: A 14-Year Nationwide Cohort Study
Source: J Clin Med. 2025 Dec 31;15(1):313. doi: 10.3390/jcm15010313 (PMC12786907; doi:10.3390/jcm15010313)
Supplement: Supplementary file 1 [file jcm-15-00313-s001.zip › jcm-4059308-supplementary.pdf]

Table S1. International Classification of Disease (10th edition) Clinical Modification (ICD 10-CM) codes used to define study population, outcomes, and comorbidities

Diseases	ICD 10-CM codes or RID codes or procedure/device/test code	Diagnosis definition
Amyloidosis	E85 or V121	Primary diagnosis, with ≥1 hospitalization or ≥1 outpatient visit
Heart failure	I50	Primary diagnosis or secondary diagnosis, with ≥1 hospitalization or ≥1 outpatient visit
Cardiomyopathy	I42	≥1 hospitalization or ≥1 outpatient visit
Atrial fibrillation	I48	≥1 hospitalization or ≥1 outpatient visit
Pacemaker insertion	Procedure code: O0203, O0204<br>and device code: G8201, G8202, G8203, G8204, G8205	≥1 hospitalization or ≥1 outpatient visit
BNP	C3681, C3682, C3682001, C3682002, C3682003, C3682004, C3682007, C7204, CZ204, CZ208, CZ208001, CZ208002, CZ208003, CZ208004, CZ208007, D4061010, D4061011, D4061012, D4061013, D4061014, D4061017, D4061020, D4061021, D4061022, D4061023, D4061024, D4061027, D4062010, D4062011, D4062012, D4062013, D4062014, D4062017, D4062020, D4062021, D4062022, D4062023, D4062024, D4063010, D4063017	Test code presence (BNP or NT-proBNP)
Echocardiography	E9431, E9432, E9433, E9434, E9435, E9436, EA431, EA432, EA433, EA434, EB430, EB431, EB432, EB433,	Test code presence

	EB434, EB435, EB436, EB610, EB611	
Cardiac MRI	HI240, HI224, HI124, HI324, HJ124, HI540, HI140	Test code presence
Ischemic heart disease	I20-I25	Primary or secondary diagnosis, with ≥1 hospitalization or ≥2 outpatient visits
Acute myocardial infarction	I21, I22	Primary or secondary diagnosis, with ≥1 hospitalization or ≥2 outpatient visits
Hypertension	I10-I13, I15	≥1 hospitalization or ≥2 outpatient visits with corresponding diagnosis under the prescription of antihypertensive drugs
Diabetes	E11-E14	≥1 hospitalization or ≥2 outpatient visits with corresponding diagnosis under the prescription of antidiabetic drugs
Dyslipidemia	E78	Primary or secondary diagnosis, with ≥1 hospitalization or ≥2 outpatient visits
Ischemic stroke	I63, I64	Primary or secondary diagnosis, with ≥1 hospitalization or ≥2 outpatient visits, accompanied by a prescription for brain CT (test codes: HA441, HA451, HA461, HA851) or brain MRI (test codes: HE101, HA201, HA301, HE401, HE501)
Chronic obstructive pulmonary disease	J43-J44	Primary or secondary diagnosis, with ≥1 hospitalization or ≥2 outpatient visits
Chronic kidney disease	N02-N08, N11, N21, N14, N18, N19, N26, N158, N159, N160, N162, N163, N164, N168, Q612, Q613, Q615, Q619, E102, E112, E132, E142, I120, M300, M313, M319,	Primary or secondary diagnosis, with ≥1 hospitalization or ≥2 outpatient visits

	T858, T859, Z992	
Multiple myeloma	C90	Primary or secondary diagnosis, with ≥1 hospitalization or ≥2 outpatient visits
Lumbar spinal stenosis	M4806	Primary or secondary diagnosis, with ≥1 hospitalization or ≥2 outpatient visits
All cancer	C00-C97	Primary or secondary diagnosis, with ≥1 hospitalization or ≥2 outpatient visits

BNP = B-type natriuretic peptide; CT = computed tomography; ICD 10-CM = International Classification of Disease 10th edition Clinical Modification; MRI = magnetic resonance imaging; NT-proBNP = N-terminal pro–B-type natriuretic peptide; RID = rare intractable disease.

Table S2. Clinical outcomes based on the number of cardiac amyloidosis-defining criteria met in the propensity score-matched population

Outcomes	No. of CA criteria	Population	No. of events	Event rate (%)	Person-year	Incidence rate, n/1000 person-year	After matching (crude)	
							HR (95% CI)	*P*-value
All-cause mortality								
	0	1,010	374	37.0	4,455.5	83.9	1 (ref.)	
	1	656	265	40.4	1,895.4	139.8	1.363 (1.162–1.598)	0.0001
	2	265	127	47.9	849.5	149.5	1.518 (1.239–1.858)	<0.0001
	≥3	89	44	49.4	386.9	113.7	1.290 (0.944–1.764)	0.1102
Cardiovascular mortality								
	0	1,010	40	4.0	4,455.5	9.0	1 (ref.)	
	1	656	34	5.2	1,895.4	17.9	1.698 (1.068–2.699)	0.0251
	2	265	21	7.9	849.5	24.7	2.394 (1.406–4.078)	0.0013
	≥3	89	6	6.7	386.9	15.5	1.633 (0.692–3.857)	0.2631

CA = cardiac amyloidosis.

Figure S1. Standardized mean differences across baseline characteristics before and after propensity score matching

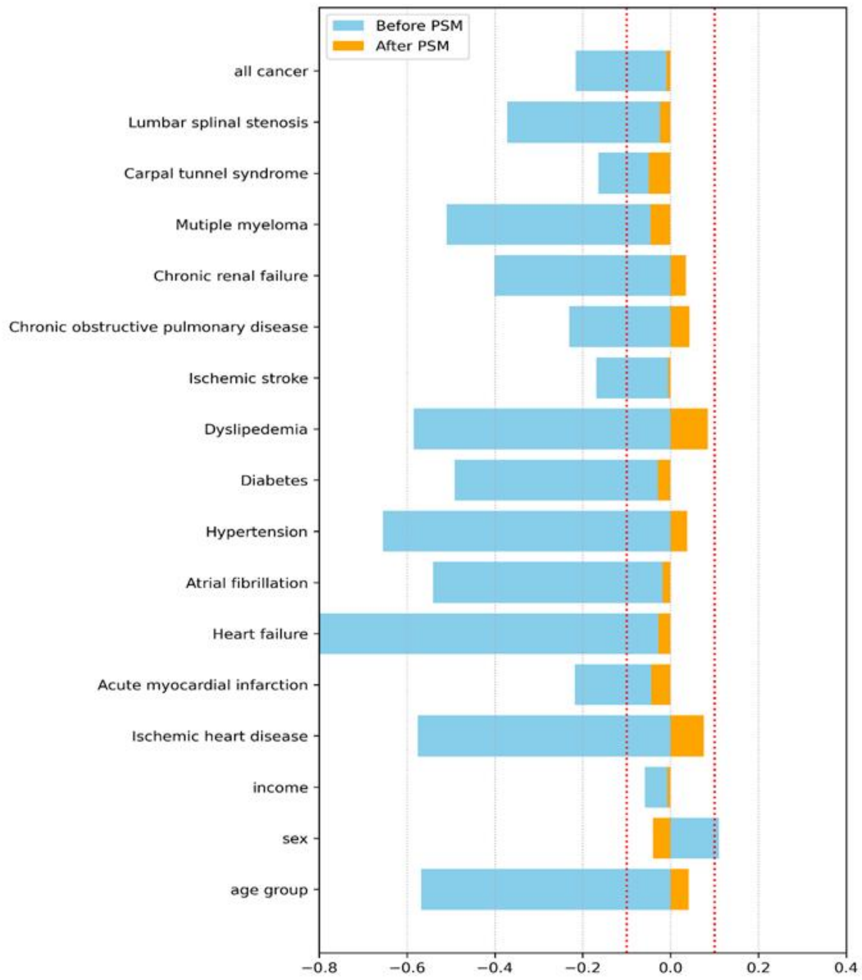

PSM = propensity matching.

Blue bars indicate standardized mean differences (SMDs) before matching, and orange bars indicate SMDs after matching. All covariates

showed SMDs below 0.2 after matching, indicating adequate balance between groups.
